# Comparison between silicon photomultiplier-based and conventional PET/CT in patients with suspected lung cancer—a pilot study

**DOI:** 10.1186/s13550-019-0504-y

**Published:** 2019-09-09

**Authors:** Johan Economou Lundeberg, Jenny Oddstig, Ulrika Bitzén, Elin Trägårdh

**Affiliations:** 1 Department of Clinical Physiology and Nuclear Medicine, Skåne University Hospital, 221 85 Lund, Sweden; 2 Radiation Physics, Skåne University Hospital, Lund, Sweden; 3 Wallenberg Center for Molecular Medicine, Lund University, Lund, Sweden

**Keywords:** Lung cancer, Digital PET, Analogue PET, TNM stage, BSREM

## Abstract

**Background:**

Lung cancer is one of the most common cancers in the world. Early detection and correct staging are fundamental for treatment and prognosis. Positron emission tomography with computed tomography (PET/CT) is recommended clinically. Silicon (Si) photomultiplier (PM)-based PET technology and new reconstruction algorithms are hoped to increase the detection of small lesions and enable earlier detection of pathologies including metastatic spread. The aim of this study was to compare the diagnostic performance of a SiPM-based PET/CT (including a new block-sequential regularization expectation maximization (BSREM) reconstruction algorithm) with a conventional PM-based PET/CT including a conventional ordered subset expectation maximization (OSEM) reconstruction algorithm. The focus was patients admitted for ^18^F-fluorodeoxyglucose (FDG) PET/CT for initial diagnosis and staging of suspected lung cancer. Patients were scanned on both a SiPM-based PET/CT (Discovery MI; GE Healthcare, Milwaukee, MI, USA) and a PM-based PET/CT (Discovery 690; GE Healthcare, Milwaukee, MI, USA). Standardized uptake values (SUV) and image interpretation were compared between the two systems. Image interpretations were further compared with histopathology when available.

**Results:**

Seventeen patients referred for suspected lung cancer were included in our single injection, dual imaging study. No statically significant differences in SUV_max_ of suspected malignant primary tumours were found between the two PET/CT systems. SUV_max_ in suspected malignant intrathoracic lymph nodes was 10% higher on the SiPM-based system (*p* = 0.026). Good consistency (14/17 cases) between the PET/CT systems were found when comparing simplified TNM staging. The available histology results did not find any obvious differences between the systems.

**Conclusion:**

In a clinical setting, the new SiPM-based PET/CT system with a new BSREM reconstruction algorithm provided a higher SUV_max_ for suspected lymph node metastases compared to the PM-based system. However, no improvement in lung cancer detection was seen.

## Background

Lung cancer is one of the most common cancers in the world with about two million new cases each year [1]. Early detection and correct staging are fundamental for optimal treatment and prognosis [2]. TNM staging in lung cancer is based on the location and extent of the primary tumour (T); the presence of mediastinal, intrapulmonary or hilar lymph node metastases (N); and the presence of distant metastases (M) [3, 4]. Positron emission tomography with computed tomography (PET/CT) with ^18^F-fluorodeoxyglucose (FDG) is recommended in the initial diagnosis and staging of patients with suspected lung cancer and can help to discriminate between malignant and non-malignant lymph nodes [5]. PET/CT can also evaluate the prognosis of small lung cancer tumours (< 3 cm) using standardized uptake values (SUV) [6].

Recently, a new generation of PET systems was introduced based on silicon (Si) photomultiplier (PM) technology. SiPM-based PET technology is hoped to increase the diagnostic accuracy via a higher spatial resolution and higher sensitivity [7–9]. Concurrently, a new block-sequential regularization expectation maximization (BSREM) reconstruction algorithm was introduced that allows for fully convergent iterative reconstruction leading to higher image contrast with limited noise [10–15]. Thus, this combination of new hardware and software could lead to early detection of pathologies including metastatic spread. In 2016, Knopp et al. [16] compared how a digital and an analogue PET/CT system perform looking at malignant and metastatic lung lesions in 20 patients. However, to the best of our knowledge, a comparison including lymph node lesions has not been previously published in patients with suspected lung cancer.

The objective of this study was to compare a SiPM-based time-of-flight system (Discovery MI; GE Healthcare, Milwaukee, WI, USA) using the BSREM reconstruction algorithm (Q.clear; GE Healthcare, Milwaukee, WI, USA) with a conventional PM-based time-of-flight system (Discovery 690; GE Healthcare, Milwaukee, WI, USA) using the ordered maximum likelihood expectation maximization (OSEM) with regard to simplified metabolic TNM staging and lesion SUV_max_ in patients undergoing ^18^F-FDG PET/CT due to suspected lung cancer.

## Method

### Study population

Patients with symptoms that might be due to lung cancer (such as bloody coughs or new dyspnea in a smoker or former smoker of at least 40 years of age) are referred according to a standardized care plan in Sweden. We included patients referred to Skåne University Hospital for PET/CT according to this standardized care plan. Patients were included when double examinations were possible and a previous diagnostic CT was available. Patients below 18 years of age, pregnant, or with a previous history of lung cancer were excluded. The study was approved by the Regional Ethical Review Board in Lund (#2016/417) and the Radiation Protection committee at Skåne University Hospital Sweden (#SSFo2016-018). All patients provided written informed consent.

### Patient preparation

Patients fasted for 4 h prior to the examination. The B-glucose levels were measured before injection of 4 MBq/kg ^18^F-FDG. After injection, the patients rested in a heated room for 60 min.

### PET/ CT scanning

Image acquisition was performed on the SiPM-based time-of-flight scanner GE Discovery MI (DMI) and the conventional PM-based time-of-flight scanner GE Discovery 690 (D690). Half of the patients were first examined on the DMI system, and the other half were first examined on the D690 system. A low-dose CT (120 kVp, 30–160 mA, noise index of 45, and slice thicknesses of 2.5 and 3.75 mm for the DMI and D690, respectively) was used without administration of oral or intravenous contrast agent on both systems for attenuation correction and anatomic correlation before the PET acquisition. An adaptive statistical iterative CT-reconstruction technique was used for the DMI, and a filtered back projection reconstruction was used for the D690.

The DMI and D690 have an axial field of view of 20 cm and 15.7 cm, respectively. An overlap of 24% between bed positions was used. PET data from the inguinal region to the base of the skull were acquired. For the D690, the PET acquisition time was 120 s per bed position; the OSEM reconstruction algorithm was 3 iterations and 12 subsets with a 5-mm Gaussian post filter and a 192 × 192 matrix (pixel size 3.6 × 3.6 mm^2^, slice thickness 3.3 mm). For the DMI, the PET acquisition time was 90 s per bed position, and the BSREM reconstruction algorithm (Q.clear; GE Healthcare, Milwaukee, WI, USA) had a *β* value of 550 with a 256 × 256 matrix (pixel size 2.7 × 2.7 mm^2^, slice thickness 2.8 mm). During installation of the DMI, signal-to-noise ratio (SNR) in the liver was used to select the acquisition time and reconstruction parameters in order to yield an equal SNR between the DMI and the D690. Due to different sensitivity for the systems (6.9 cps/kBq and 14 cps/kBq for the D690 and DMI, respectively), the same SNR was obtained at different acquisition times. All reconstructions included time-of-flight and point spread function corrections. Respiratory gating was not used. Both PET-CTs are cross-calibrated to the same dose calibrator, and the calibration is validated monthly in a SUV control with phantoms.

### Image interpretation

Images were interpreted by two nuclear medicine physicians with 6 and more than 10 years of experience with PET/CT in lung cancer, respectively. All suspected malignant lesions were recorded to determine a simplified metabolic TNM stage. The metabolic TNM stages were defined as: a primary tumour (T+ or T−), suspected malignant intrathoracic lymph nodes (N+ or N−), or distant metastases (M+ or M−). The reviewers evaluated the scans in a blinded fashion without knowledge of the PET/CT scanner, clinical history, sex, age, and patient outcome. The latest diagnostic CT or referral information was available on request for all patients when the findings were difficult to interpret on the PET examination, for example to better determine if lesions were malignant or inflammatory. Information was given in 7 cases. All PET/CT images were evaluated on an Extended Brilliance Workstation version V4.5.3.40140 (Philips Healthcare, Cleveland, OH, USA).

The histology results from endobronchial ultrasound with real-time-guided transbronchial needle aspiration (EBUS-TBNA), surgery, and biopsies were collected from the patients’ medical records when available and compared to the results from the simplified TNM staging (9 patients).

### Quantitative analysis

>SUV_max_ was measured in the primary tumour and a maximum of 3 intrathoracic suspected malignant lymph nodes with variable sizes. Lymph nodes from different intrathoracic regions were preferred, if available, to allow for representative measurements. Subgroup analysis of SUV_max_ in lymph nodes smaller than 10 mm was performed (short-axis measured on an axial CT slice).

In addition, the SUV_mean_ was measured in a circular region-of interest (ROI) in the blood pool in the left atrium (ROI 900–1000 mm^2^) and in the mid-region of the right liver lobe (ROI 3000 mm^2^), respectively_._ If liver metastases were present, the ROI was moved to an area with no detectable metastases. The SUV_ratio_ was defined as lesion SUV_max_/physiological SUV_mean_ and was calculated using blood pool SUV_mean_ and liver SUV_mean_ as physiological references. The SNR was calculated by dividing liver SUV_mean_ by SUV_standard deviation_ of the liver ROI.

### Statistical analysis

Differences in SUV between the SiPM-based and the PM-based PET/CT systems were compared using the Wilcoxon signed-rank test. Continuous variables are presented as mean ± SD. A *P* value of less than 0.05 was considered statistically significant. Bland-Altman analyses were used to assess the agreement between the two systems. The statistical software GraphPad Prism7 version 7.00 was used.

## Results

A total of 17 patients were included (5 women, 12 men; 69 ± 5 years) (see Table 1 for patient information). The injected ^18^F-FDG dose was 3.99 ± 0.15 MBq/kg. The accumulation time for the first scan was 58 ± 2 min, and the delay to the second examination was 31 ± 6 min. Accumulation time for scans at the DMI system was 75 ± 15 min and for the D690 system was 72 ± 18 min. The B-glucose level before ^18^F-FDG injection was 114 ± 27 mg/dL.

**Table 1:** Patient characteristics and scanning setup

Case	Age (decade)	Sex	Weight (kg)	Height (cm)	B-glucose (mg/dL)	^18^F-FDG-dose (MBq)	Accumulation time DMI (min)	Accumulation time D690 (min)
1	6th	M	101	176	169	399	57	87
2	7th	M	94	171	101	375	98	56
3	7th	M	54	169	114	212	57	81
4	7th	F	70	158	115	281	84	56
5	6th	M	65	178	97	236	59	86
6	6th	F	64	162	123	250	59	85
7	6th	M	94	182	97	368	57	95
8	6th	M	67	173	85	277	58	85
9	6th	M	73	176	132	296	88	55
10	7th	F	49	165	92	194	61	87
11	6th	M	89	183	103	353	57	88
12	7th	M	83	178	87	333	55	85
13	6th	F	37	168	128	164	88	61
14	5th	M	62	176	97	249	107	57
15	7th	M	71	169	180	282	89	62
16	7th	M	74	168	101	294	87	57
17	6th	F	80	165	96	318	91	59

### Quantitative image analysis

No statistically significant difference was found between the PET/CT systems regarding SNR (Table 2). The SUV_mean_ in the liver was significantly different, but only slightly lower for the DMI compared to the D690; no statistical difference was found in the blood pool.

**Table 2:** Physiological, signal-to-noise ratio, and all lesion SUV parameters

SUV parameters (mean ± SD)	Discovery 690	Discovery MI	*P* value	Change (%)
Signal-to-noise ratio	10.9 ± 2.6	10.2 ± 2.2	0.53	− 6
Liver SUV_mean_	2.4 ± 0.5	2.2 ± 0.3	*0.01	− 10
Blood pool SUV_mean_	1.8 ± 0.3	1.7 ± 0.3	0.22	− 7
Primary lesions SUV_max_	10.9 ± 8.2	10.3 ± 7.1	0.29	− 6
Primary lesions to blood pool SUV_ratio_	6.6 ± 5.2	6.3 ± 4.6	0.84	− 5
Primary lesions to liver SUV_ratio_	5.0 ± 3.9	4.9 ± 3.5	0.84	− 2
Lymph node SUV_max_	6.4 ± 5.5	7.0 ± 5.2	*0.026	10
Lymph node to blood pool SUV_ratio_	3.8 ± 3.5	4.2 ± 3.0	0.11	12
Lymph node to liver SUV_ratio_	2.8 ± 2.6	3.2 ± 2.3	*0.021	17

*Indicating a statistically significant difference

Of the 17 patients, 13 had a suspected malignant primary lesion (seen on either of the systems) with a mean long axis at 45 ± 25 mm and short axis 33 ± 20 mm measured on a trans-axial CT-slice. For the primary lesions, no statistically significant difference was found between the PET/CT systems regarding SUV_max_, lesion to blood SUV_ratio_, and lesion to liver SUV_ratio_.

**Fig. 1:**
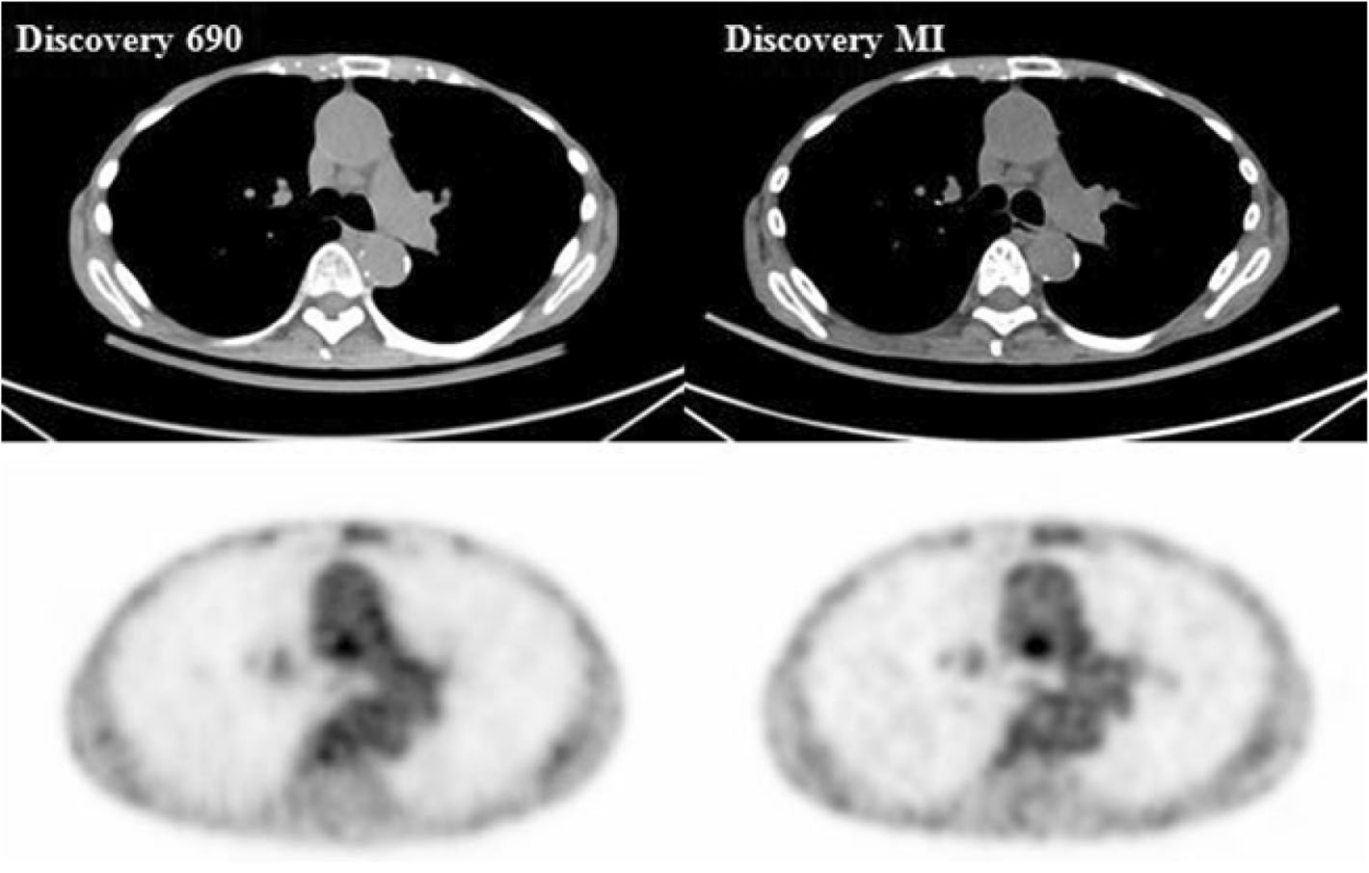
Axial PET and CT images. An example of axial PET and CT images of a mediastinal lymph node with a SUV_max_ of 2.7 on the Discovery 690 and 3.6 on the Discovery MI

A total of 22 metabolically suspected malignant intrathoracic lymph nodes (seen on either of the systems) with a mean long axis at 12.3 ± 4.2 mm and a short axis at 8.2 ± 2.7 mm were examined. The lesion SUV_max_ and lesion to liver SUV_ratio_ were significantly higher on the DMI compared to the D690 (Table 2); the lesion to blood pool SUV_ratio_ did not differ significantly. Figure 1 shows a representative example of differences in SUV_max_ between the DMI and the D690 for a mediastinal lymph node.

**Fig. 2:**
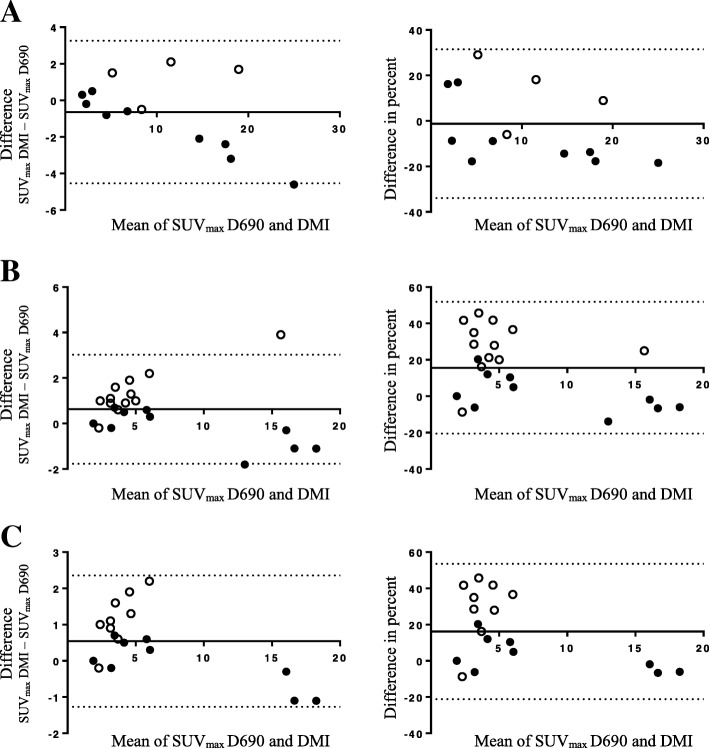
Bland-Altman plots comparing SUV_max_ between the Discovery 690 (D690) and Discovery MI (DMI). Absolute differences (left column) and differences in percent (right column) between SUV_max_ on D690 and DMI (*y*-axis) vs. the average of D690 and DMI (*x*-axis). White circles indicate lesions from patients first examined on the D690 and black circles first examined on the DMI. The DMI produced higher values for SUV_max_ in all lymph nodes (**b**) and lymph nodes less than 10 mm on short axis (**c**) but not in primary lesions (**a**)

Furthermore, subgroup analysis was done on 18 of the 22 metabolically suspected malignant intrathoracic lymph nodes with a short axis < 10 mm (mean long axis at 10.9 ± 3.1 mm and short axis 7.1 ± 1.4 mm). SUV_max_ in these lymph nodes was significantly higher on the DMI compared with the D690 (Table 3). The extra-thoracic metastases were not studied with SUV measurements due to a very low number of suspect lesions (6 lesions in 2 patients). Bland-Altman plots of primary lesion SUV_max_ and lymph node metastases for the two systems are shown in Fig. 2. The difference between the two PET/CT systems (expressed as absolute difference and difference in percent) was higher for low SUV_max_.

**Table 3:** SUV parameters in lymph nodes < 10 mm

SUV parameters (mean ± SD)	Discovery 690	Discovery MI	*P* value	Change (%)
Lymph node lesion SUV_max_	5.8 ± 5.5	6.3 ± 4.9	0.026*	9
Lymph node lesion to blood pool SUV_ratio_	3.4 ± 3.5	3.8 ± 2.9	0.17	11
Lymph node lesion to liver SUV_ratio_	2.5 ± 2.7	2.9 ± 2.2	0.054	14

*Indicating a statistically significant difference

### Image interpretation

Overall there was a good consistency between the cameras comparing the simplified TNM-stage, but a different interpretation was seen in three cases (Table 4).

**Table 4:** Metabolic TNM stage based on the two PET examinations

Case	Staging DMI	Staging D690	Change in staging	Histology results
1	T+N−M−	T+N+M−	YES	Epithelial cancer (S)
2	T+N+M−	T+N+M−	NO	Colon cancer (B)
3	T+N−M−	T−N−M−	YES	Not done
4	T−N−M−	T−N−M−	NO	Adenocarcinoma (S, E)
5	T+N−M−	T+N−M−	NO	Suspect malignant cells (B)
6	T+N+M−	T+N+M−	NO	Non-small cell lung cancer (E)
7	T+N+M+	T+N+M+	NO	Adenocarcinoma (B)
8	T+N+M−	T+N+M−	NO	No malignant cells (Br)
9	T+N+M−	T+N+M−	NO	Non-small cell lung cancer (Br)
10	T+N−M−	T+N−M−	NO	Non-small cell lung cancer (S,E)
11	T+N−M+	T+N−M+	NO	Non-small cell lung cancer (Br)
12	T−N−M−	T+N+M−	YES	No malignant cells (E,B)
13	T+N+M−	T+N+M−	NO	Small cell lung cancer (E)
14	T−N−M−	T−N−M−	NO	Not done
15	T+N+M−	T+N+M−	NO	Epithelial cancer (Br)
16	T−N−M−	T−N−M−	NO	Not done
17	T−N−M−	T−N−M−	NO	Not done

In case of malignant and non-malignant findings, only the malignant histology results are shown above

*S* surgery, *B* biopsy, *E* EBUS-TBNA, *Br* bronchoscopy with brush/fluid sample

In the first case, the physicians identified an additional lymph node on the D690 that was not seen on the DMI leading to up-scaling of the TNM-stage. However, the histology results showed no malignant lymph nodes indicating a false-positive result on the D690. The second case showed a suspect primary tumour on the DMI that was not seen on the D690. No histology results were available because the treating physician chose to monitor the patient clinically. In the last case, the physicians identified a suspect primary tumour and two lymph nodes on the D690; neither was seen on the DMI. Histology results from EBUS-TBNA showed no malignant cells indicating a false positive on the D690; however, a few reactive cells were seen. All three patients were first examined on the DMI. Neither PET/CT system detected a malignant primary lesion and malignant lymph nodes (adenocarcinoma) in one patient resulting in a false-negative result. Of the 4 cases where no histology was performed, 3 patients were monitored clinically with control CTs within 1–2 years and 1 patient was deemed not needing further follow-ups after the PET/CT examination.

The PET results (both suspected malignant and none malignant findings) were compared to histology results from the EBUS-TBNA, surgery, and biopsies as reference methods (Tables 5 and 6). The histology results from bronchoscopy with brush/fluid samples are not shown due to the uncertain origin of these cells. Similar findings were shown regarding lymph nodes, but two additional false-positive lymph nodes were seen on the Discovery D690.

**Table 5:** Primary lesion—histology compared to metabolic TNM stage

Discovery 690		Discovery MI	
True positive	False positive	True positive	False positive
4	1	4	0
False negative	True negative	False negative	True negative
1	0	1	1

The histology results from the EBUS-TBNA, surgery, and biopsy’s as gold standard compared with PET results

**Table 6:** Lymph node—histology compared to metabolic TNM stage

Discovery 690		Discovery MI	
True positive	False positive	True positive	False positive
2	2	2	0
False negative	True negative	False negative	True negative
2	20	2	22

The histology results from the EBUS-TBNA, surgery, and biopsy’s as gold standard compared with PET results

## Discussion

To evaluate the clinical value, we compared the whole PET/CT systems including both hardware and software. Several studies have analysed how the BSREM algorithm affects the images with better image quality, tumour conspicuousness, and image sharpness compared to OSEM [10] as well as a lower background noise and a higher SUV_max_. Teoh et al. [17] showed that SUV_max_ was higher in lung lesions smaller than 10 mm using BSREM but it did not improve the differentiation between malignant and benign lesions. Other studies have compared the hardware of the SiPM-based and the conventional PM-based PET/CT systems in phantom studies using the same reconstruction algorithms [18, 19]. Wagatsuma et al. [19] showed that the Discovery MI had a better contrast compared to the Discovery 710 when the same reconstruction algorithms were used. In 2016, Knopp et al. [16] compared how a digital PET/CT system and an analogue perform looking at malignant and metastatic lung lesions. However, to the best of our knowledge, a comparison including lymph node lesions has not been previously investigated in patients with suspected lung cancer.

Differences in SNR in the liver were not expected since the acquisition time and reconstruction parameters for the DMI were selected to give the same SNR as for the D690 at the installation.

Even though statistically significant, the difference between the PET/CT systems regarding liver SUV_mean_ was relatively small. It cannot be explained by the difference in accumulation time [20] and might be due to a random effect. In the blood pool, we saw only a marginal decrease in SUV that was not statistically significant which might indicate that blood SUV_mean_ is more reliable than liver SUV_mean_ as suggested by Hofheinz et al. [21].

No significant difference in SUV_max_ was seen for the primary tumours. This is most likely because the primary tumours were relatively large leading to less impact on the different reconstruction algorithms. The higher SUV_max_ in suspected lymph node metastases (mean of 12 × 8 mm) for the DMI is most likely due to the BSREM algorithm that leads to an increased SUV in small lesions [17].

The new SiPM-based PET/CT systems have a higher spatial resolution and a higher sensitivity and thus may increase the detectability of malignancy in PET/CT [7, 17]. Although the SiPM technology together with BSREM reconstruction yielded higher SUV values for metabolically malignancy suspected lymph nodes, we could not verify that the new system will detect more malignant lesions. Of our three cases with differences in the metabolic TNM stage, two were an up-stage due to findings on the PM-based system and one was up-staged on the SiPM-based system. These three patients were first examined on the DMI so our findings are not explained by the order in which the PET/CT systems were used. We found only very minor differences in a further analysis using histology results from EBUS-TBNA, surgery, and biopsies. This was contrary to our initial beliefs since previous studies indicated an increased sensitivity and decreased specificity [7, 17]. Thus, we have not verified that the new digital PET/CT system will improve the ability to detect malignancy in a clinical setting where both systems had been optimized individually regarding image quality and time efficiency. This might be because the D690 is a relatively new system including time-of-flight and point-spread-function. A larger difference in image quality and SUV is to be expected when comparing SiPM-based PET/CT to older generations of PET-scanners without time-of-flight and point-spread-function correcting capabilities. The greater axial FOV (20 vs 15.7 cm for the DMI and the D690, respectively) and the high sensitivity permit faster image acquisition (90 vs 120 s/bed for the DMI and the D690, respectively). Generally, our experience is that the total PET acquisition time is almost half that for the D690. A short acquisition time potentially reduces problems with patient motion and bladder filling and would allow more patients to be examined per day.

The difference in ^18^F-FDG accumulation time may have resulted in differences between the two PET scans in each patient. The randomized scanning order for the systems yielded a similar mean accumulation time (75 ± 15 min for the DMI and 72 ± 18 min for the D690 system) implying that this probably did not affect the results for the whole group. Optimization of the reconstruction algorithm settings can be further improved as more studies and clinical experiences are gathered. Furthermore, the small sample size, lack of respiratory gating, and few patients with biopsy-proven disease limits the strength of our study.

## Conclusion

This clinical study found that a SiPM-based PET/CT system together with a BSREM reconstruction algorithm yielded higher SUV_max_ for suspected malignant lymph nodes compared to the conventional PM-based system. However, no improved ability to detect lung cancer was seen. Further larger studies are thus warranted to investigate if the new SiPM-based PET/CT systems as well as improved reconstruction algorithms will impact diagnosis, clinical management, and patient outcome. It was possible to use a shorter acquisition time for the Discovery MI with preserved diagnostic result.

## Abbreviations

^18^F-FDG: ^18^F-fluorodeoxyglucose
BSREM: Block-sequential regularization expectation maximization
CT: Computed tomography
D690: Discovery 690
DMI: Discovery MI
EBUS-TBNA: Endobronchial ultrasound with real-time guided transbronchial needle aspiration
OSEM: Ordered subset expectation maximization
PET: Positron emission tomography
ROI: Region of interest
SiPM: Silicon photomultiplier
SNR: Signal-to-noise ratio
SUV: Standardized uptake value
SUV_max_: Maximum standardized uptake value
SUV_mean_: Mean standardized uptake value
